# C-855-02. Austere Burn Care: Efficacy of Educating Non-Burn Providers Where Burn Centers Do Not Exist

**DOI:** 10.1093/jbcr/irag033.086

**Published:** 2026-04-06

**Authors:** Michael L Cheatham, Melissa Martin, Jessica Lyn Burger, Joany McDougall, Sasha Thew

**Affiliations:** Warden Burn Center at Orlando Health - Orlando Regional Medical Center, Orlando, FL; Vanderbilt University Medical Center, Nashville, TN; Cleveland Clinic, Barberton, OH; London Health Sciences Centre, London, Ontario; London Health Sciences Centre, London, Ontario

## Abstract

**Introduction:**

Ninety percent of burn injuries occur in low- or middle-income countries where burn centers are rare and most burn care is delivered by local healthcare providers. Following a mass casualty burn event which killed an estimated 200 victims and injured an additional 300, the World Health Organization (WHO) requested the creation of a four-day comprehensive multidisciplinary burn care and rehabilitation conference to educate healthcare providers on providing ongoing burn care for these victims. This course has subsequently been revised, condensed to two days, and taught twice to non-burn providers who work in developing countries without the ability to transfer patients to a burn center. An accompanying 130-page manual details state-of-the art burn care and rehabilitation concepts with education for providing this care under austere conditions with limited resources.

**Methods:**

Participants in the three burn care and rehabilitation courses received pre- and post-conference surveys. Participants were asked to rate their understanding of burn care concepts on a scale of 1 to 100. They were also asked to identify key burn management principles that they felt were important to their understanding of burn care and rehabilitation. The survey results for the three courses were combined and analyzed using descriptive statistics to confirm the efficacy of the educational training and guide future course revisions.

**Results:**

Eighty-five healthcare providers from a variety of disciplines have participated in the austere burn care and rehabilitation course. Participant understanding of burn management concepts increased from 50% pre-conference to 82% post-conference (*p*<.0001). The five most important topics of interest to participants were: how to provide burn care with limited resources, burn dressings, surgical debridement, pain control, and rehabilitation therapy.

**Conclusions:**

Burn injuries are common worldwide, but many healthcare providers in developing countries lack both burn management and rehabilitation education as well as the ability to refer burn patients to a burn center. For these providers, burn care and rehabilitation education, oriented to available local resources, is essential to improve burn victim outcome. A comprehensive two-day course oriented to providing burn care and rehabilitation using limited resources significantly improves understanding of burn management concepts in non-burn providers.

**Applicability of Research to Practice:**

Burn injuries and mass casualty incidents are common in the developing world, but tertiary burn centers are not. Burn education of local healthcare providers is essential to improve burn victim outcome.

**Funding for the Study:**

N/A.

